# A mobile phone-based care model for outpatient cardiac rehabilitation: the care assessment platform (CAP)

**DOI:** 10.1186/1471-2261-10-5

**Published:** 2010-01-28

**Authors:** Darren L Walters, Antti Sarela, Anita Fairfull, Kylie Neighbour, Cherie Cowen, Belinda Stephens, Tom Sellwood, Bernadette Sellwood, Marie Steer, Michelle Aust, Rebecca Francis, Chi-Keung Lee, Sheridan Hoffman, Gavin Brealey, Mohan Karunanithi

**Affiliations:** 1 Cardiology Program, The Prince Charles Hospital, Brisbane, Qld, Australia; 2 The Australian E-Health Research Centre, CSIRO, The Royal Brisbane and Women's Hospital, Brisbane, Australia; 3 Primary and Community Health Services Metro North Health Service District, Brisbane, Qld, Australia

## Abstract

**Background:**

Cardiac rehabilitation programs offer effective means to prevent recurrence of a cardiac event, but poor uptake of current programs have been reported globally. Home based models are considered as a feasible alternative to avoid various barriers related to care centre based programs. This paper sets out the study design for a clinical trial seeking to test the hypothesis that these programs can be better and more efficiently supported with novel Information and Communication Technologies (ICT).

**Methods/Design:**

We have integrated mobile phones and web services into a comprehensive home- based care model for outpatient cardiac rehabilitation. Mobile phones with a built-in accelerometer sensor are used to measure physical exercise and WellnessDiary software is used to collect information on patients' physiological risk factors and other health information. Video and teleconferencing are used for mentoring sessions aiming at behavioural modifications through goal setting. The mentors use web-portal to facilitate personal goal setting and to assess the progress of each patient in the program. Educational multimedia content are stored or transferred via messaging systems to the patients phone to be viewed on demand. We have designed a randomised controlled trial to compare the health outcomes and cost efficiency of the proposed model with a traditional community based rehabilitation program. The main outcome measure is adherence to physical exercise guidelines.

**Discussion:**

The study will provide evidence on using mobile phones and web services for mentoring and self management in a home-based care model targeting sustainable behavioural modifications in cardiac rehabilitation patients.

**Trial registration:**

The trial has been registered in the Australian New Zealand Clinical Trials Registry (ANZCTR) with number ACTRN12609000251224.

## Background

Cardiovascular disease (CVD) is the most common cause of death in Australia, accounting for 34% and 39% of male and female deaths, respectively in Australia in 2007 [1]. Ischemic heart disease is the leading cause of mortality and accounts for 17.4% of all deaths in both males and females attributable to life-style related risk-factors [1]. The total burden of this disease is likely to increase given the increase in morbid obesity and diabetes and the growing number of elderly patients in whom these diseases are more common.

A number of modifiable environmental and patient specific factors increase the chance of developing coronary heart disease [2]. These include smoking, high blood cholesterol, physical inactivity, diabetes, high blood pressure, obesity and depression, social isolation and lack of social support. Cardiac rehabilitation has provided an avenue for reducing cardiovascular risk in patients into the future by positively influencing these factors [3]. The eventual goal of the program is to engage patients with permanent life-style change regarding their health behaviours, i.e. behavioural change. There is good evidence for the effectiveness of cardiac rehabilitation particularly from studies of patients with myocardial infarction and following coronary revascularization [4].

Unfortunately low levels of patient participation (14-43% after myocardial infarction) are consistently reported in Australia, France, the UK, New Zealand and the USA [5,6]. These low patient participation rates are reported to be a consequence of low levels of service provision, referral and invitation, and of poor uptake by patients. The lack of referrals is a major barrier in the uptake of cardiac rehabilitation. In an Australian audit only 11% of acute chest pain patients were formally referred to phase II cardiac rehabilitation across all centres [7]. Patients with the best rates of participation in cardiac rehabilitation programs have tended to be male, middle-aged and diagnosed with uncomplicated myocardial infarction. Those most likely to benefit such as those with significant functional impairment, older patients, women and ethic groups are the least likely to participate in a program. However a number of other very practical factors such as difficulties with transportation, lack of interest, dislike of classes/hospitals, work or domestic commitments, rural residence, location and accessibility and car parking all influence utilization of cardiac rehabilitation [8]. Hence, approaches to improve rates of utilization of cardiac rehabilitation needs to be investigated. Home based models are considered as a feasible alternative to avoid various barriers related to care centre based programs and several studies have reported these models to be as effective as the traditional models [9].

The current state of mobile phone communication and technology provides not only the capacity but an especially attractive media option to support home-based health and chronic disease management programs. As a technology platform, it integrates sufficient computing power, user interface, memory, and communication capabilities to run applications needed for personal health management. From an individual user's perspective it has become a personal appendage to be considered as a sufficiently personal and trusted device to store personal messages, to conduct daily errands (such as carry out bill payments or monetary transactions while on the move, and it is carried even while exercising or doing some other daily tasks. More importantly, the mobile phone is available while making our daily health related behavioural choices such as going for a walk. Mobile phones are attractive tools from health service providers' perspective because they have the capacity to deliver multimedia communication and information for feedback at a personal level to the patient in combination with a very high penetration rate in most countries (e.g. in Australia 99% and in many countries significantly higher than for example broadband internet penetration). The mobile phone is a relatively well accepted device, which makes it potentially a non-discriminating service media. Some earlier studies have successfully used mobile phone applications to wellness management [10,11]. This paper will describe the methods, integrated solution and clinical trial protocol to evaluate a cardiac rehabilitation care model delivered using a mobile phone platform.

## Methods/Design

### Applied technologies

We have developed a care model that efficiently utilises mobile phones, internet and communication technologies as a means to deliver rehabilitation services to outpatients participating in a 6 week program in their own homes or wherever they are. The mobile phones are used for monitoring of exercise and other health data as well as recording patients' self observations on their health related behaviour. All the data is synchronised daily to a WellnessDiary Connected (WDC) portal on a remote server. The phones include a 3G phone plan provided without cost to the patient which covers all the required communication and data transfer services. The patients receive motivational and educational multimedia materials and SMS messages through the phone. They discuss and set goals with their Mentor on weekly phone consultations during the programme. The Mentors access the patient's recent data on the WDC portal prior to the phone consultation to facilitate and personalise feedback and goal setting with each patient. Video calls on the mobile phone can be used if face-to face discussion is preferred.

Two software tools are installed and used on the mobile phone: 1) Step Counter (SC) and 2) WellnessDiary (WD). The SC uses the phone's inbuilt accelerometer to count the user's steps, walking time and stepping intensity. The SC will launch automatically each morning to register any steps taken during the day. The patients are instructed to carry the mobile phone with them throughout the day to enable the to automatically capture of their steps and also enable them to contact a support person if needed. Patients are provided with scales and blood pressure monitor for daily measurements. The WD allows entries of other relevant health parameters such as weight, fat %, exercise (other than steps), blood pressure, stress, tiredness, sleep, eating, smoking and alcohol use. The diet information can be collected via the Wellness Diary entries and additionally by taking photos of the meals by using the phone's digital camera. The patients and Mentors can view their entries and trend graphs directly on the phone or on the WDC portal. The platform is used to capture information that is not currently objectively measured in the home environment.

We hypothesize that these technologies offer effective health and care management tools for both the patients and Mentors.

**Figure 1:**
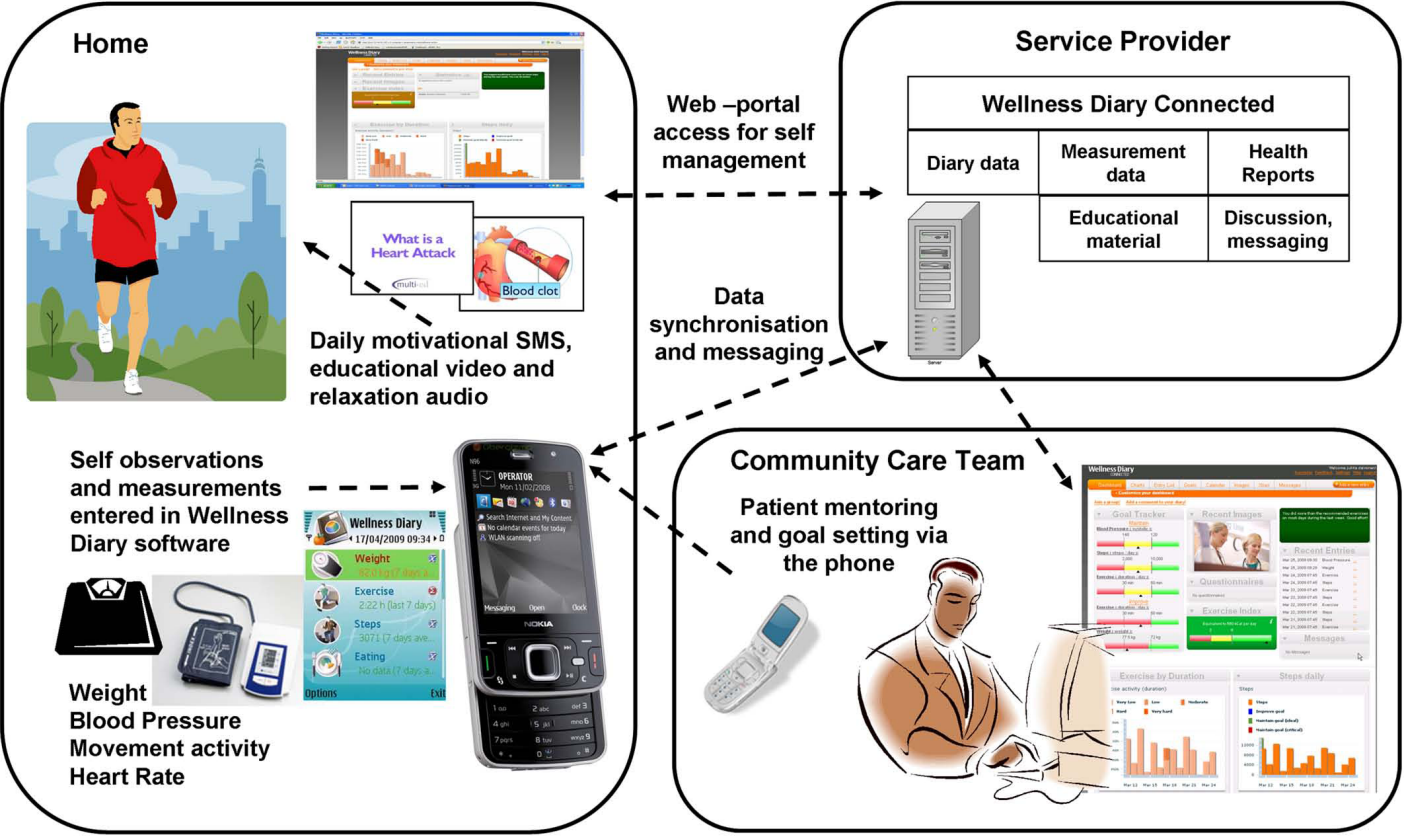
**CAP System diagram**. The mobile phone acts as the communication medium through which a) the Community Care Tam provides mentoring and goal setting, b) daily motivational messages, educational videos and relaxation audio are sent, and c) self observations and measurements are entered to the Wellness Diary application. All data is synchronised and stored on a daily basis to a Wellness Diary Connected portal on a remote server.

The CAP technology platform diagram is presented in Figure. 1.

### Care model

Our rehabilitation program structure was developed according to the Australian national guidelines [12] to cover all the components of a comprehensive cardiac rehabilitation program. The patient will be enrolled through an initial assessment at the community care centre including: baseline measurements; training to use the different technologies and software; education on avoiding risks; and correct exercise levels; and briefing on details such as contact persons. The patient also meets his/her personal Mentor who will set the first exercise and lifestyle goals at the start of the home program.

**Figure 2:**
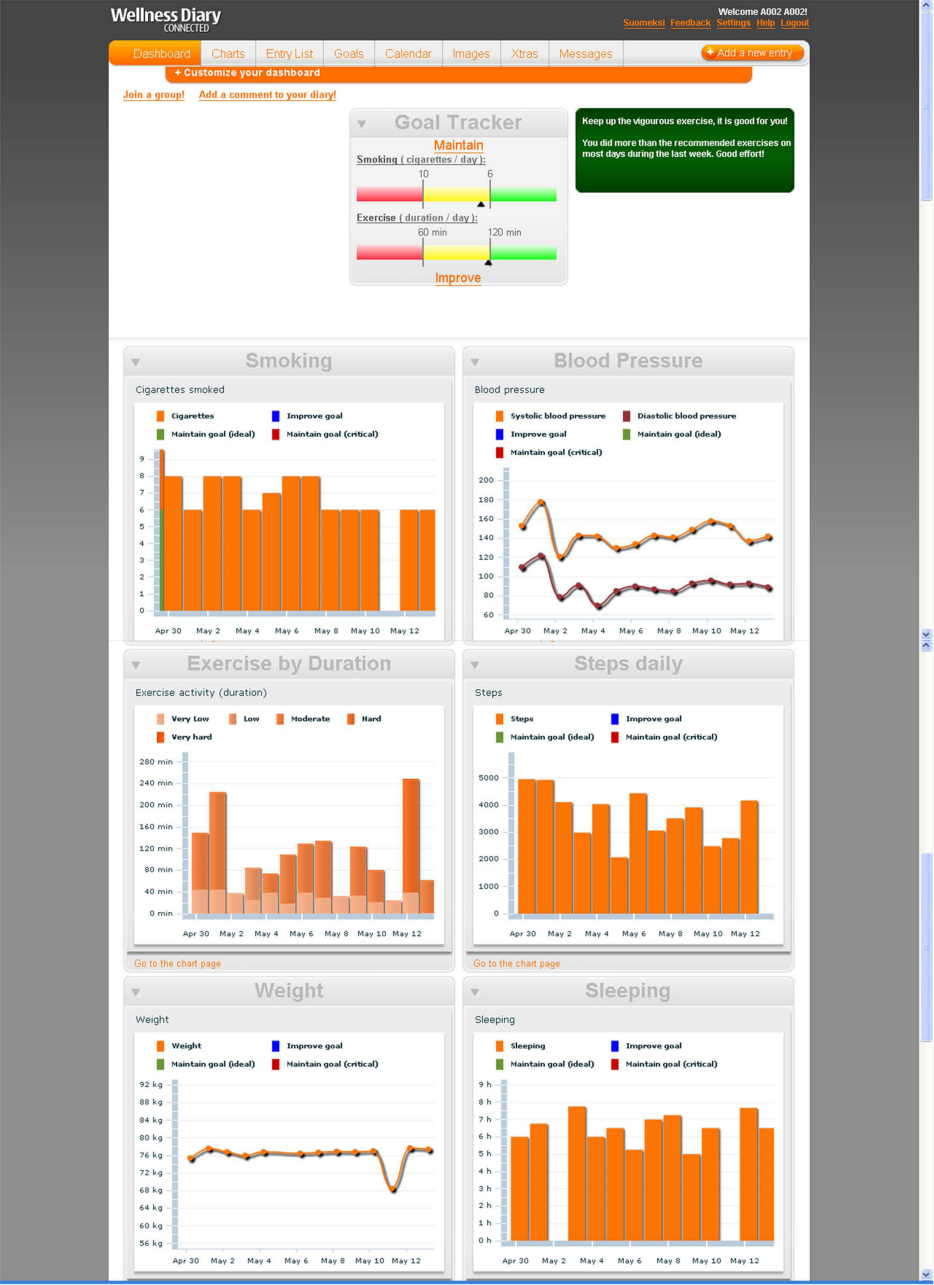
**Wellness Diary Connected web-portal, Dashboard view**. Arbitrarily chosen patient's data on the Wellness Diary Connected web-portal at the end of the 6 week rehabilitation program. The top of the screen shows the Goal Tracker bar graph used to set and follow personal goals for reducing smoking and increasing exercise duration. The Smoking chart shows the patients self-recorded daily amount of cigarettes (6-8/day). The Blood Pressure and Weight charts depict the measured values during the past 2 weeks. Exercise by Duration chart shows the total amount of exercise (average 120 min/day) and the Steps daily chart the patient's walking activity (2000-5000 steps/day) measured with the phone's inbuilt accelerometer. Sleeping chart shows the self-reported sleeping time (5-8 h/night).

The 6 week program consists of weekly Mentoring sessions focussing in different themes on healthy lifestyle and behaviours, risk factor management and education. The Mentor will discuss the patient's progress in comparison to the set goals and assist in setting the new goals on exercise and behavioural modifications for the following week. Both the patient and Mentor will document the agreed goals. The patients will receive daily SMS messages that contain short motivational messages on the topics of the weekly themes. The phone includes additional educational multimedia material on each topic and relaxation audio files that the patient can listen to any time and anywhere. The daily exercise program at home consists of light to moderate levels of walking as the main mode of physical activity. The patients will be individually assessed by the Mentor and the amount and levels of exercise and other behaviours are gradually modified through SMART (Simple, Measurable, Achievable, Realistic and Time-bound) goal setting. The target is to engage the patient in at least 30 minutes of moderate level of physical activity on most of the days of the week as recommended by the Heart Foundation [13]. The step counts and exercise entries on the mobile phone that are synchronised with the WDC service provide a tool for the Mentor to measure patients' walking and other exercise and to assess their adherence to the goals and guidelines. Figure 2 shows an example WellnessDiary and Step Counter software on the mobile phone. These are self management tools for the patients to observe their own progress against the personal goals set with the Mentor.

The mentoring phase of the rehabilitation program ends after 6 weeks with a post-assessment meeting at the community centre. The patient's progress and current status will be evaluated and a continuation plan for the next 6 months follow-up period will be designed with the Mentor. The mentoring sessions over the video and telephone calls will not be continued, but the patient is encouraged to use the mobile phone and portal tools for self management.

### Study design

**Figure 3:**
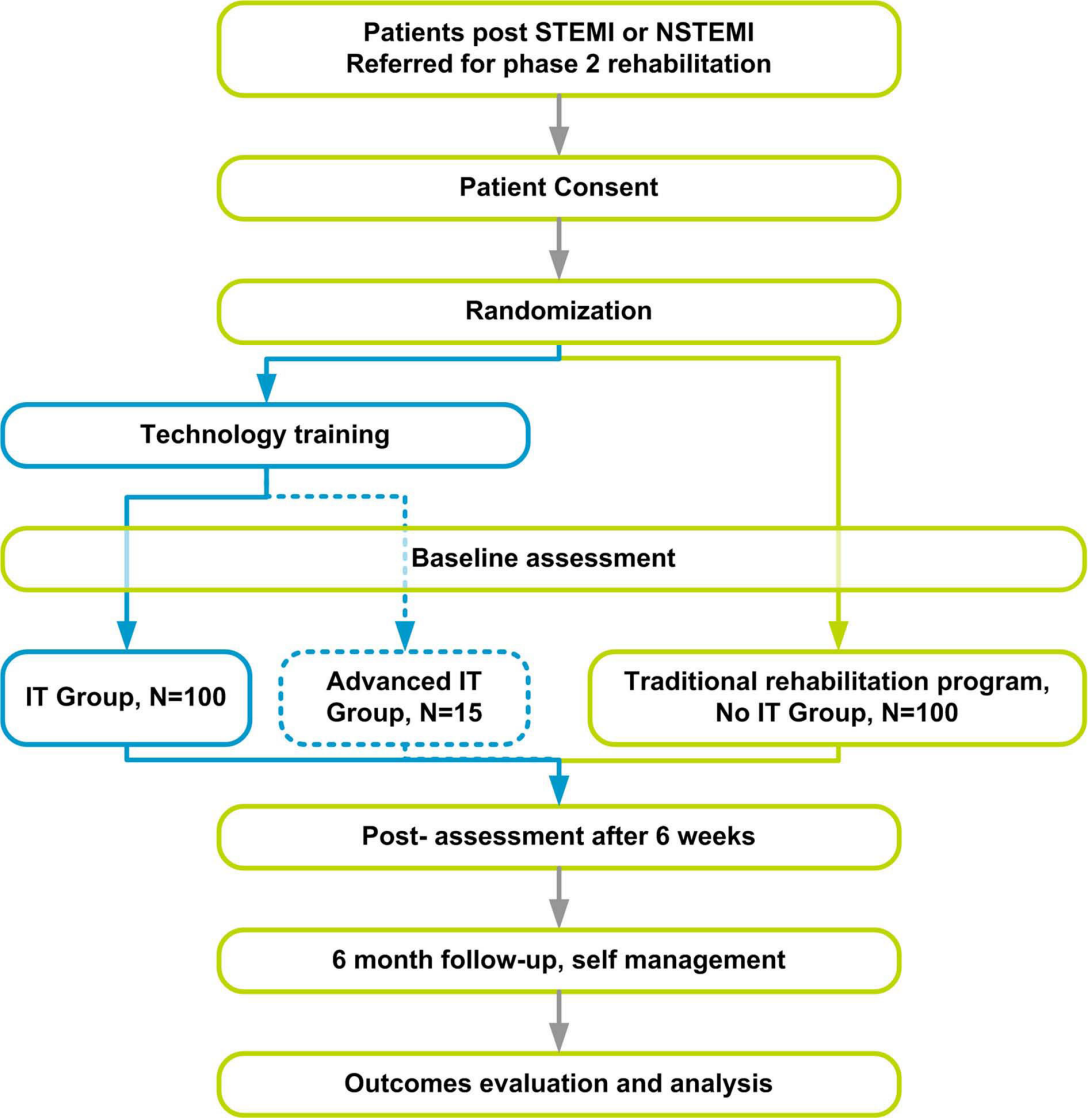
**RCT Design block diagram**. The study is a prospective randomized comparison of a traditional cardiac rehabilitation program versus a mobile phone based cardiac rehabilitation program that utilises information and communication technology. The study includes patients with STEMI or NSTEMI referred for Phase 2 rehabilitation.

The Study is an open randomized controlled trial of Information Technology (IT) enabled home based rehabilitation program utilizing mobile phones (IT Group) compared to traditional community based rehabilitation program (No IT Group). The study design is displayed in Figure 3. The trial will be conducted in Primary & Community Health Services of Metro North Health Service District of Queensland Health. The work is jointly funded by The Australian E-Health Research Centre (AEHRC), CSIRO and Queensland Health, Australia. The study has been approved by the ethics committee of the Redcliffe-Caboolture hospital and the patient recruitment commenced in April 2009.

The goal is to provide evidence that the new home-based care model is a viable alternative and provides health outcomes equivalent to the traditional care model of cardiac rehabilitation services and is equally cost efficient. Positive results would imply possibilities to roll out the program to a large scale program as an alternative care model in addition to the traditional program.

A smaller subgroup (Advanced IT Group) of the home-based rehabilitation arm will trial additional ambulatory monitoring technologies such as heart rate and movement activity measurement devices. The aim is to develop methods to assess the patients' heart function during home-exercises and monitor their physiological status and development during the rehabilitation program.

The study is designed as a non inferiority trial with a minimum of 100 patients planned to be enrolled in each arm of the study. The primary outcome measure is adherence to physical activity guidelines assessed at the baseline, end of the 6 week program and 6 month follow up period. A number of secondary outcomes are also being assessed and a cost effectiveness analysis is being performed. The outcome measures and the corresponding measurement tools are listed in Table 1.

**Table 1:** Outcomes measured at baseline, after 6 weeks and 6 months.

Outcome Measures	Measurement tools
**Primary Outcome Measure**	
Adherence to physical activity guidelines.	Active Australia Survey [17] Walking activity measured with a pedometer over 7 days 6 minute walk test

**Secondary Outcome Measures**	

Risk factors:	
Body Mass Index (BMI)	Measured at the cardiac rehabilitation centre and Queensland Medical Laboratories according to standard procedures and recorded in a Cardiac Rehabilitation Assessment tool
blood pressure	
smoking	
alcohol intake	
Full Blood Count (FBC), lipids, HbA1c, Haemoglobin	

Psychological Functioning	Kessler 10 [18]

Nutrition Status	Diet Habits Questionnaire [19]

Quality of Life	European Quality of Life-5 Dimensions (EQ-5D) Health Outcome questionnaire [20] The Seattle Angina Questionnaire [21]

Medication Compliance	Self report

Satisfaction & Usability	Questionnaire for the patients and clinical staff

Morbidity and Mortality	Unplanned re-admission and Mortality obtained from Queensland Health Hospital Based Computer Information System (HBCIS)

Process Indicators:	
Costs	Staff time reports on Care Continuum Suite (CCS) system, projected equipment and facility costs are collected from the hospital's financial database, other technology costs are calculated from the project's financial records or estimated from the current market values
Drop-out rates in the control and intervention groups, numbers/percentage of people who did/did not consent to participate in the trial	Trial recruitment spreadsheet
Median time return-to-work/return to work	Patient self-report

A cost-benefit analysis will be performed throughout the study. Our goal is to provide evidence that the developed model is a cost-efficient, effective and viable alternative to a traditional institution-based cardiac rehabilitation. We will use an Activity Based Costing Model developed by Nexus Online Pty Ltd [14]. The model will address fixed and variable costs for an existing gym based program and the proposed technology-enabled home-based program on a per patient basis. The costing model includes both direct and indirect costs related to the delivery of a six week program, with re-assessment after 6 months.

A previous study by Arrigo et. al. [15] detected a significant 33% difference in exercise adherence with patients attending a home-based care versus standard care (73% and 40% respectively). Based on this result, we used the method described by Kirby et al [16] to calculate the number of participants to be able to show a 20% improvement of physical exercise adherence at 80% power and 5% significance level. The result was that approximately 100 subjects were required in each group and the recruitment of 143 participants in each group to allow for an estimated attrition rate of 30%. The patient allocation will be carried out through permuted block randomization to ensure a balance in the patients to each group. Varying block sizes of 4, 6, and 8 were used and both the allocation within the blocks and the block order were randomized to create the allocation table. The Project Officer who obtains the patient consent will randomize them by using a sequence of sealed envelopes containing the allocation for each patient. The randomization table is concealed from the Project Officer and created and maintained by the AEHRC.

## Discussion

We have developed an alternative home-based care model for outpatient cardiac rehabilitation based solely on mobile technologies and internet services. The work includes a re-design of the rehabilitation process as well as development of the technology platform, which is trialled in a randomized study in the community care setting. The main strengths of the model include true mobility and reliance on existing mobile phone and networking technologies which allows potentially cost efficient implementation in various geographical settings. This study will provide evidence on the utility, validity and cost-efficiency of the developed care model. It will also investigate the ability of the relatively aged cardiac patients to use given technologies in real life settings, which is currently an open issue and considered as a potential barrier for applying modern technology tools in outpatient care. We may be able to identify a sub-group of patients that prefer and benefit from a home-based care over the traditional model.

The technology platform presented in this paper may offer a highly scalable solution for various home-based care models. The platform offers advantages in terms of affordability, flexibility and access with the wide spread use of mobile phones and web services in the community. It is likely that some of the features used in this study and currently available only in high-end phones will be standard in majority of the future phone models reducing the overall costs. The main operating costs for the health care provider, other than staff time, are phone calls, messaging services and web-portal license fees, which are likely to be less expensive than the face-to-face consultations requiring travelling.

Our study will provide comprehensive evidence on using mobile phones and web services for mentoring and self management in a home-based care model targeting sustainable behavioural modifications in cardiac rehabilitation patients.

## Competing interests

The authors declare that they have no competing interests.

## Authors' contributions

DW, AS, AF and MK designed the study setup and initiated the project. AS and MK proposed the technology setup which was integrated with the practical care model developed with TS, BSel, MS, MA, RF, CL, SH and GB. The trial process and outcome measurement tools have been implemented and partly developed by KN, CC and BS who also co-ordinate the conduct of the trial. DW wrote the manuscript outline, Background and Study Design sections. AS wrote the Applied Technologies and Care Model sections and finalized the manuscript. All authors read and contributed to the final manuscript.

## Pre-publication history

The pre-publication history for this paper can be accessed here:

http://www.biomedcentral.com/1471-2261/10/5/prepub
